# ICTV Virus Taxonomy Profile: Xinmoviridae 2023

**DOI:** 10.1099/jgv.0.001906

**Published:** 2023-10-26

**Authors:** Stephen Sharpe, Sofia Paraskevopoulou

**Affiliations:** 1 Hawkesbury Institute for the Environment, Western Sydney University, Penrith, NSW 2751, Australia; 2 Genome Competence Center (MF1), Robert Koch Institute, Berlin, Germany

**Keywords:** arthropod, ICTV Report, *Mononegavirales*, taxonomy, *Xinmoviridae*

## Abstract

*Xinmoviridae* is a family of viruses with negative-sense RNA genomes of 9–14 kilobases. Xinmovirids typically infect beneficial and pest insects but their host range has not yet been investigated systematically and hence may be broader. This is a summary of the International Committee on Taxonomy of Viruses (ICTV) Report on the family of *Xinmoviridae*, which is available at ictv.global/report/xinmoviridae.

## Virion

Xinmovirids (viruses in the family *Xinmoviridae*) are only known from metagenomics studies. Virions have not yet been visualized and structural proteins have not been studied.

## Genome

**Fig. 1.**
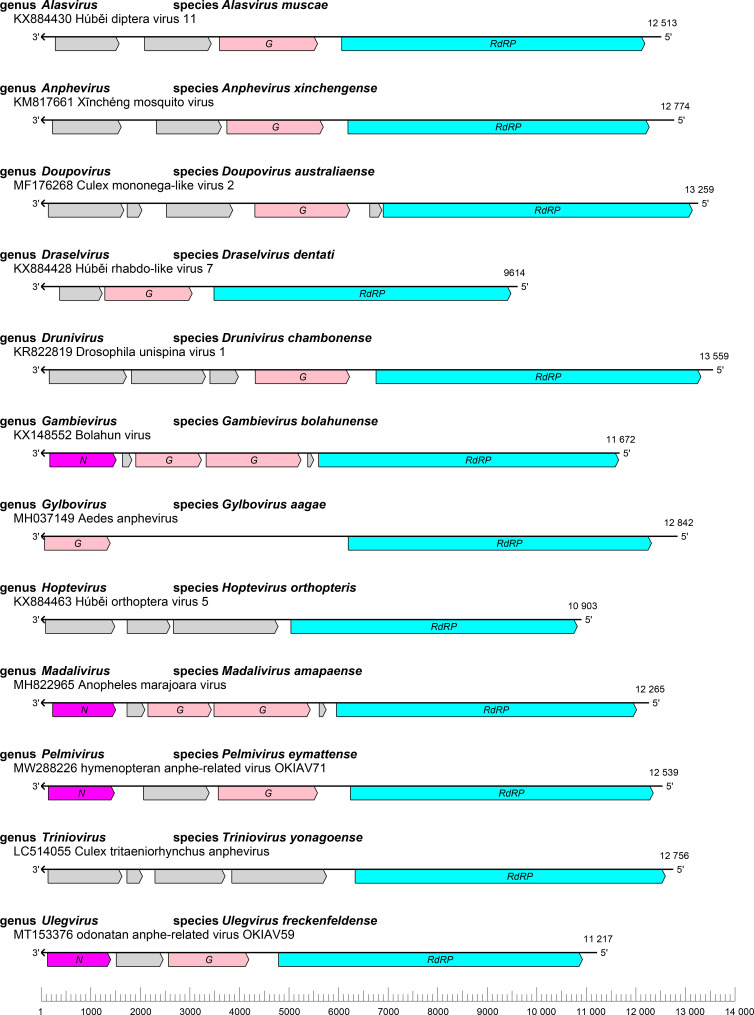
Genome organization of a representative virus from each genus in the family *Xinmoviridae*. ORFs are coloured according to their predicted protein function: *N*, nucleoprotein gene (magenta); *G*, glycoprotein gene (pink); *RdRP*, RNA-directed RNA polymerase gene (cyan).

Xinmovirids have negative-sense genomes of 9–14 kilobases with three to six ORFs (Table 1, Fig. 1) [1] that encode at least three structural proteins that have been identified via comparison with proteins encoded by other mononegavirals: a glycoprotein (G), a nucleoprotein (N), and an RNA-directed RNA polymerase (RdRP) [2].

**Table 1.** Characteristics of members of the family *Xinmoviridae*

Example	Drosophila unispina virus 1 (KR822819), species *Drunivirus chambonense*, genus *Drunivirus*
Virion	Unknown
Genome	9–14 kb of negative-sense RNA
Replication	Unknown
Translation	Unknown
Host range	Arthropoda
Taxonomy	Realm *Riboviria*, kingdom *Orthornavirae*, phylum *Negarnaviricota*, class *Monjiviricetes*, order *Mononegavirales*: >11 genera and >13 species

## Replication

Xinmovirids are only known from metagenomics studies. Their replication cycles have not yet been studied.

## Pathogenicity

Pathogenicity is unknown, but based on their host spectrum, xinmovirids likely have an effect on arthropod fitness.

## Taxonomy

**Fig. 2.**
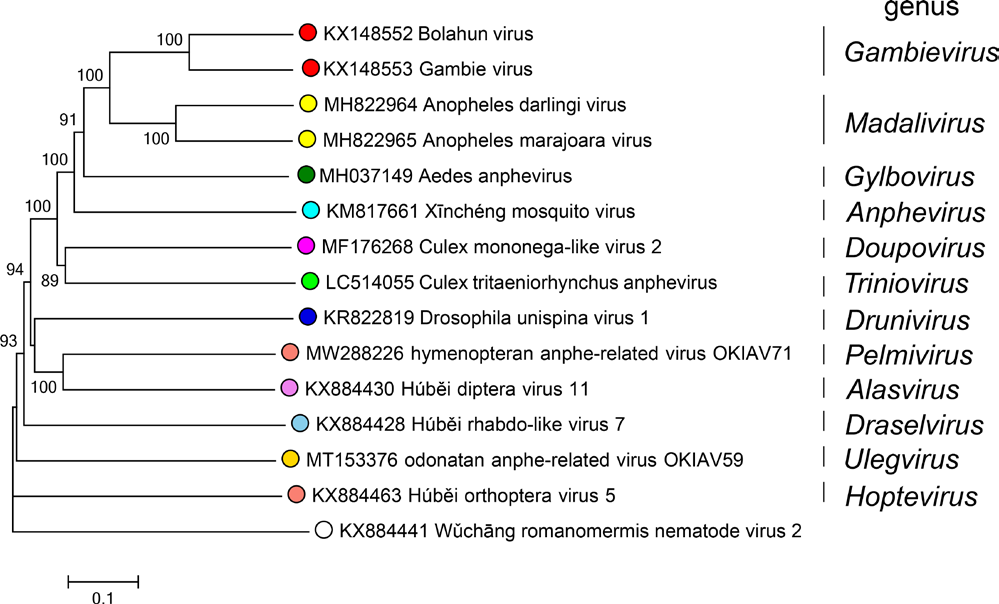
Phylogenetic tree of xinomovirid RdRP amino acid sequences. The virus Wǔchāng romanomermis nematode virus 2 (family *Lispviridae*) was used as an outgroup. Circles at tips are coloured by genus.

Current taxonomy: ictv.global/taxonomy. The family *Xinmoviridae* includes >11 genera and >13 species (Fig. 2). Hosts include arthropods, specifically insects from the orders Diptera, Hymenoptera, and Orthoptera. Related, unclassified viruses have been discovered in mosquitos [3,6] and tephritid fruit flies [7,8].

## Resources

Full ICTV Report on the family *Xinmoviridae*: ictv.global/report/xinmoviridae.

## Abbreviations

G: glycoprotein
N: nucleoprotein
RdRP: RNA-directed RNA polymerase
